# tRNA copy number and codon usage in the sea cucumber genome provide insights into adaptive translation for saponin biosynthesis

**DOI:** 10.1098/rsob.210190

**Published:** 2021-11-10

**Authors:** Chengzhang Liu, Jianbo Yuan, Xiaojun Zhang, Songjun Jin, Fuhua Li, Jianhai Xiang

**Affiliations:** ^1^ CAS and Shandong Province Key Laboratory of Experimental Marine Biology, Center for Ocean Mega-Science, Institute of Oceanology, Chinese Academy of Sciences, Qingdao 266071, People's Republic of China; ^2^ Laboratory for Marine Biology and Biotechnology, Qingdao National Laboratory for Marine Science and Technology, Qingdao 266237, People's Republic of China

**Keywords:** tRNA, adaptive translation, saponin biosynthesis, cytochrome P450, UDP-glycosyltransferases

## Abstract

Genomic tRNA copy numbers determine cytoplasmic tRNA abundances, which in turn influence translation efficiency, but the underlying mechanism is not well understood. Using the sea cucumber *Apostichopus japonicus* as a model, we combined genomic sequence, transcriptome expression and ecological food resource data to study its codon usage adaptation. The results showed that, unlike intragenic non-coding RNAs, transfer RNAs (tRNAs) tended to be transcribed independently. This may be attributed to their specific Pol III promoters that lack transcriptional regulation, which may underlie the correlation between genomic copy number and cytoplasmic abundance of tRNAs. Moreover, codon usage optimization was mostly restrained by a gene's amino acid sequence, which might be a compromise between functionality and translation efficiency. Genes for stress responses were highly optimized for most echinoderms, while enzymes for saponin biosynthesis (LAS, CYPs and UGTs) were especially optimized in sea cucumbers, which might promote saponin synthesis as a defence strategy. The genomic tRNA content of *A. japonicus* was positively correlated with amino acid content in its natural food particles, which should promote its efficiency in protein synthesis. We propose that coevolution between genomic tRNA content and codon usage of sea cucumbers facilitates their saponin synthesis and survival using food resources with low nutrient content.

## Graphical abstract

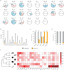

## 1. Introduction

As adaptors that link amino acids to codons in messenger RNAs (mRNAs), transfer RNAs (tRNAs) are a fundamental component of the translation machinery. tRNAs constitute up to 10% of all cellular RNA, making them the most abundant small non-coding RNAs (ncRNAs) [1]. They also outnumber other ncRNAs in terms of genomic loci, with copy numbers up to thousands in a single species [2]. Owing to the redundancy of the genetic code, each of the 20 amino acids is usually encoded by two or more synonymous codons, which vary mostly at the third position. These synonymous codons are translated by their corresponding tRNAs, which are called isoaccepting tRNAs. Although they encode the same amino acids, synonymous codons are not all used at the same frequency. This phenomenon is called codon usage bias, which is believed to be the result of mutational biases and natural selection for translation efficiency [3]. The theory is based on the observation that the speed of translation relies on the availability of the cellular tRNA pool [4], and that the more abundant cellular tRNAs are usually used more frequently in gene coding [3]*.* Unlike most other ncRNAs or coding genes, the cellular concentrations of tRNAs are highly correlated with their genomic copy numbers [4–6]. However, the mechanism underlying this correlation is not well understood. Nevertheless, the copy number of tRNA genes in the genome is often used as a proxy for their abundance in the cytoplasm [7]. In short, genomic tRNA content determines cytoplasmic tRNA composition, which in turn determines translation efficiency. The extent of compatibility between mRNA codon usage and the tRNA pool varies from gene to gene and from species to species. However, the selective pressure underlying these variations associated with translation efficiency is still poorly understood. In different yeast species, the translation efficiency of key genes in the energy metabolic process was found to evolve according to organisms' metabolic styles [4]. Nevertheless, study on adaptive evolution of translation efficiency is scarce, especially for metazoan species.

As primitive deuterostomes and close relatives of the chordates, echinoderms are ideal research models for evolutionary, molecular and developmental biology. They possess fascinating evolutionary innovations that are rare in the animal kingdom, such as a bilaterally organized embryo but a pentaradial adult body plan, a unique water vascular system and an extraordinary capability for regeneration. From the perspective of metabolomics, echinoderms are characterized as being slow moving, having a hypometabolism during aestivation, having remarkable longevity and having the ‘plant-like’ ability to produce a secondary metabolite called saponin (triterpenoid glycoside). Among the extant five echinoderm classes, sea cucumbers (Holothuroidea) are especially prolific in saponin production (also known as holothurins) [8]. More than 700 Holothuroidea saponins have been isolated in the past 60 years, and one sea cucumber species may produce more than 50 types of saponin [9,10]. Synthesized as repellents against predators, infectious fungi, protists and other parasites, saponins are accumulated in the skin, body wall and especially in the sticky Cuvierian tubules that can be discharged through the anus as a defence strategy [8]. Although highly toxic to fish, saponins are valued by humans for their appealing pharmacological potential, such as antimicrobial, anticoagulant, haemolytic, antiviral, antiparasitic and antitumour properties [10]. The sea cucumber *Apostichopus japonicus* naturally distributes along the west coast of the North Pacific Ocean. Traditionally prized as a tonic and now cultivated on a commercial scale, it is now the most economically important echinoderm and the most intensely studied species of sea cucumber.

Because differential translational efficiency can play key roles in phenotypic divergence, especially in the divergence of metabolic style [4], information about adaptation between codon usage and tRNA gene content would be indispensable for understanding the metabolic characteristics of sea cucumbers and other echinoderms. To date, a comprehensive survey of tRNA genomics has not been carried out for any echinoderms. Here, we surveyed tRNA genes in the recently published *A. japonicus* genome and analysed their correlation with codon usage in transcriptome data and amino acid content in the sea cucumber's natural food particles. Our analyses revealed that codon usage of enzymes catalysing saponin biosynthesis, the immune response to a stimulus and digestion was especially optimized for efficient translation. Moreover, the genomic tRNA content of *A. japonicus* was highly correlated with amino acid content in its natural food particles, which should help it to survive on marine debris with a very low nutritional content.

## 2. Results

### 2.1. tRNA genes in the *A. japonicus* genome

**Figure 1.**
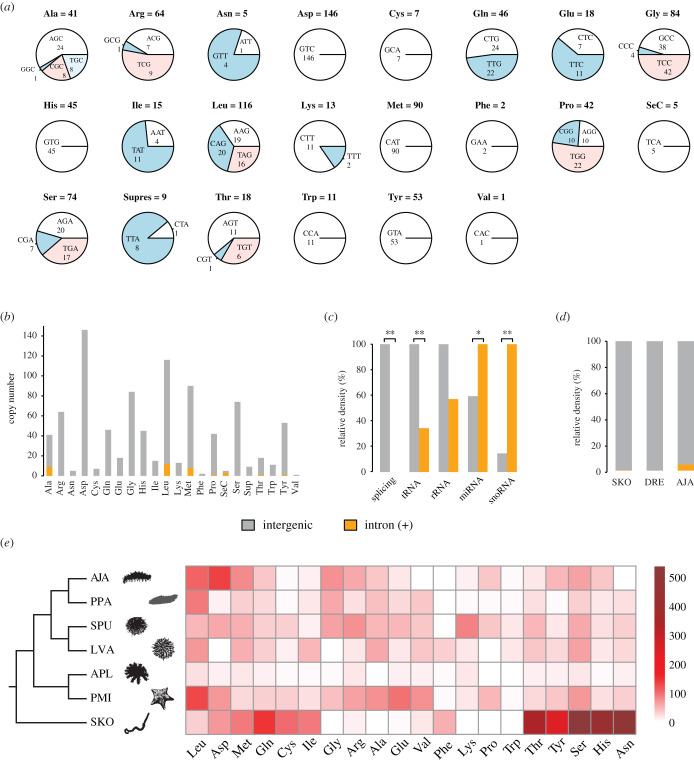
tRNA copy number in the *A. japonicus* genome. (*a*) Fraction of the different anticodons within each isoacceptor family. (*b*) tRNA copy numbers. (*c*) Distribution of non-coding RNAs according to their relation with protein-coding genes. (*d*) tRNAs are scarce in introns of coding genes in different deuterostomes. (SKO, *Saccoglossus kowalevskii*; DRE, *Danio rerio*; AJA, *A. japonicus*). (*e*) tRNA copy number in different Ambulacraria groups. (AJA, *A. japonicus*; APL, *Acanthaster planci*; SPU, *Strongylocentrotus purpuratus*; LVA, *Lytechinus variegatus*; PPA, *Parastichopus parvimensis*; PMI, *Patiria miniata*; SKO, *S. kowalevskii*).

For *de novo* annotation of tRNA genes, two strategies were assessed to distinguish tRNAs from repetitive sequences (see Methods), and a pre-filtering strategy was adopted. As a result, 1032 tRNA genes were identified, forming the largest ncRNA family for *A. japonicus* (figure 1*a*; electronic supplementary material, dataset S1). The most abundant tRNAs include those encoding anticodons for Asp (146), Leu (116), Met (90), Gly (84), Ser (74), Arg (64), etc. The rarest is tRNA for Val, which has only one copy. Although each of the 22 amino acids was represented by tRNA genes decoding at least one anticodon for that amino acid, tRNA genes for 16 specific anticodons were missing in the genome, including Arg (CCG), Asp (ATC), Cys (ACA), Gly (ACC), His (ATG), Ile (GAT), Leu (GAG), Phe (AAA), Pro (GGG), Ser (GGA, ACT), Thr (GGT), Tyr (ATA) and Val (AAC, TAC, GAC).

It is well recognized that certain types of ncRNAs tend to emerge within introns of coding genes [11,12]. Small nucleolar RNAs (snoRNAs) are a class of small RNAs that guide chemical modifications of other RNAs. miRNAs (microRNAs) are small RNAs that mediate gene regulation by inhibiting translation and/or inducing degradation of target mRNAs. For *A. japonicus*, miRNAs and snoRNAs were enriched in introns (figure 1*c*). On the other hand, only 49 loci of the 1032 tRNAs were embedded in introns. Most tRNAs were located in intergenic regions, which meant that they were transcribed independently of coding genes. tRNA density was significantly lower in introns than in intergenic regions of the genome (1 : 2.9, *p* = 0, *χ*^2^-test) (figure 1*c*). This trend was also consistent with results from other deuterostomes such as *Danio rerio* and *Saccoglossus kowalevskii* (figure 1*d*).

We compared tRNA gene composition among echinoderm lineages and their sister clade hemichordate (figure 1*e*). The results showed substantial differences among echinoderms, and even greater distinction between echinoderms and hemichordates. To understand the causes and consequences of tRNA copy number evolution, we used *A. japonicus* as a model to analyse the relation between genomic tRNA contents and codon usage of coding genes.

### 2.2. Synonymous codon usage and isoaccepting tRNA abundance co-adapted for optimal translation

**Figure 2.**
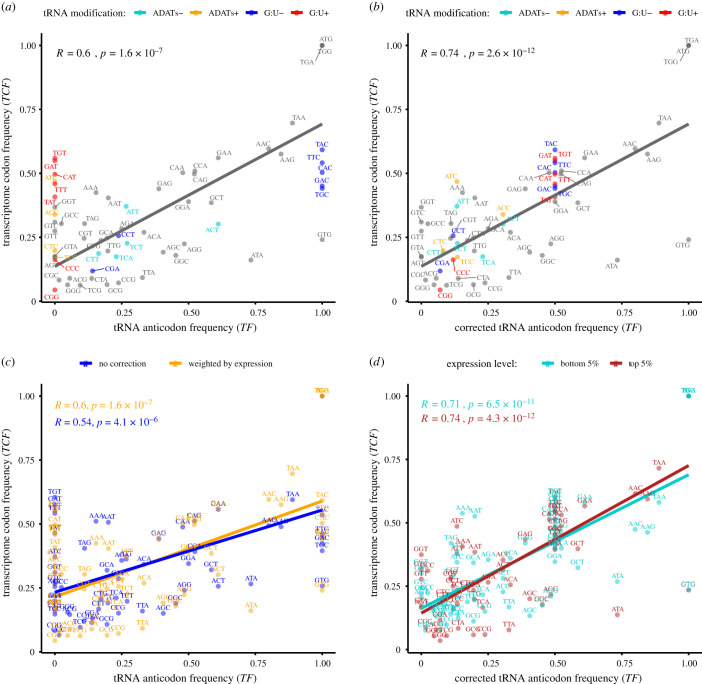
Correlation between isoaccepting tRNA gene frequencies and synonymous codon usage in the transcriptome of *A. japonicus*. (*a*) Correlations computed by weighting codons according to mRNA expression level and using the Watson–Crick base pairing rules (U : A; A : U; C : G; G : C). Notice that codons deviating from correlation were those potentially applied to G : U wobble and ADAT modifications. (*b*) Increased correlation after correction according to G : U wobble and ADAT modifications. (*c*) Weighting codons according to mRNA expression level increased the correlation only slightly. (*d*) Genes with the highest and lowest expression levels showed similar correlation levels between isoaccepting tRNA frequencies and synonymous codon usage.

**Figure 3.**
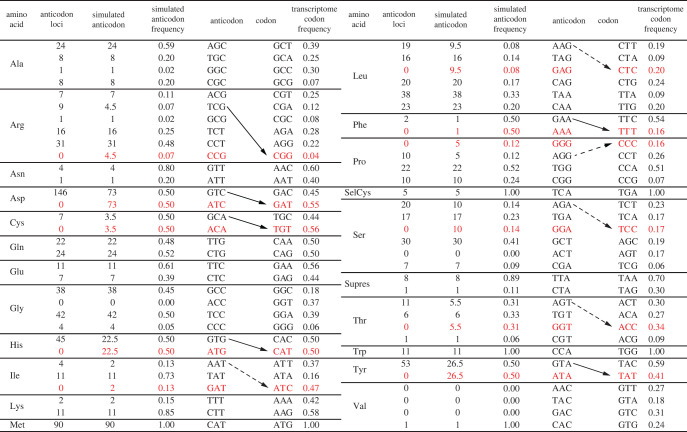
Relationships between synonymous codons and isoaccepting tRNAs suggested the existence of G : U wobbling and ADAT activity. Calculation of anticodon frequency (TF*_ij_*) and transcriptome codon frequency (TCF*_ij_*) can be found in the Methods. The loss of anticodons for which mRNA codons are abundant is shown in red. The alternative matches enabled by tRNA wobble are shown with arrows. Solid arrows indicate G : U wobble. Dashed arrows indicate pairing resulting from ADAT modifications. Data used in this figure can be found in electronic supplementary material, table S1.

Although encoding the same amino acids, synonymous codons are not all used at the same frequency. Correspondingly, the different types of tRNAs carrying a specific amino acid are called isoaccepting tRNAs. For each amino acid in all the annotated *A. japonicus* genes*,* we compared the relative usage of synonymous codons with the relative gene frequency of cognate isoaccepting tRNAs. Positive correlations were found for most of the amino acids, and a moderate correlation was observed when all the amino acids were combined (figure 2*a*). In addition, gene expression levels from transcriptome data were used to weight codon usage in the correlation calculation. As a result, the correlation increased slightly (figure 2*c*). Still, discrepancies remained for a considerable number of codons. No anticodon tRNA was found for 16 codons that showed substantial expression in the transcriptome data (figures 2*a* and 3). With further examination, we noted that some of these codons could be translated by other anticodons if G : U wobble was allowed for the third position in the codon (or base 34 of tRNA) (figure 3). Moreover, most of the remaining ‘lost’ anticodons could also be substituted by other tRNAs if tRNA-dependent adenosine deaminase (ADAT) activity was considered, which converts adenine to inosine (A-to-I editing) at base 34, enabling the match of I with U, C or A (figure 3) [13]. Taking these unconventional matches into consideration, an adjusted analysis resulted in a significantly increased positive correlation between transcriptome codon usage and tRNA copy numbers (figure 2*b*; electronic supplementary material, table S1). This observation indicates that G : U wobble and ADAT modification exists in the translation system of the sea cucumber, which increases the decoding capacity of certain tRNAs so that some other tRNA genes can be substituted or discarded.

To assess whether expression level has a crucial influence on coevolution between synonymous codons and isoaccepting tRNAs, we compared their correlation between genes with distinct expression levels. As a result, genes with the highest (top 5%) and lowest (bottom 5%) expression levels showed very similar correlation levels (figure 2*d*).

### 2.3. Co-adaption between tRNA abundances and amino acid usage is limited

**Figure 4.**
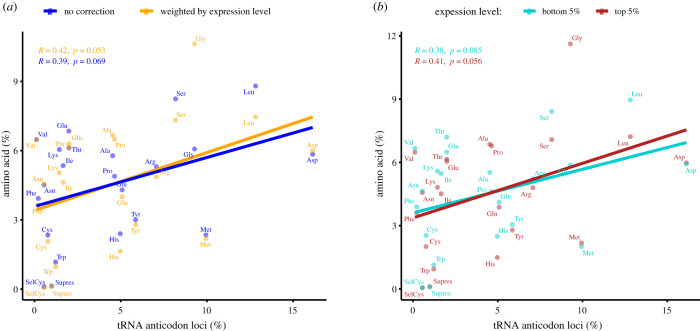
Correlation between genomic tRNA frequency and transcriptome amino acid frequency of *A. japonicus*. (*a*) Weighting amino acid frequency according to average mRNA expression level increased the correlation only slightly. (*b*) Genes with the highest and lowest expression levels showed similar correlation patterns between tRNA gene frequency and amino acid frequency.

In the preceding section, we showed that isoaccepting tRNA abundance and synonymous codon usage were highly co-adapted for optimal translation of the same amino acid. The analysis did not contain information from amino acid frequency, as the count of synonymous codons was normalized by the total count of the codons for each amino acid. For tRNAs translating different amino acids, we also found a positive correlation between the frequency of amino acids in the *A. japonicus* transcriptome and the number of genomic tRNAs (figure 4). This result was in accordance with proteomic data, because the most abundant genomic tRNAs (Asp, Gly, Arg, etc.) matched the most abundant amino acids in the body mass of the sea cucumber [14–16]. Nevertheless, in comparison with the synonymous codon level, the correlation at the amino acid level was much lower. To assess whether this lack of correlation could be attributed to the difference in gene expression level, transcriptome codons were weighted according to their expression level. However, only a slight increase in correlation was observed (figure 4*a*), which indicated that expression levels of mRNAs were not the decisive factor in this correlation.

**Figure 5.**
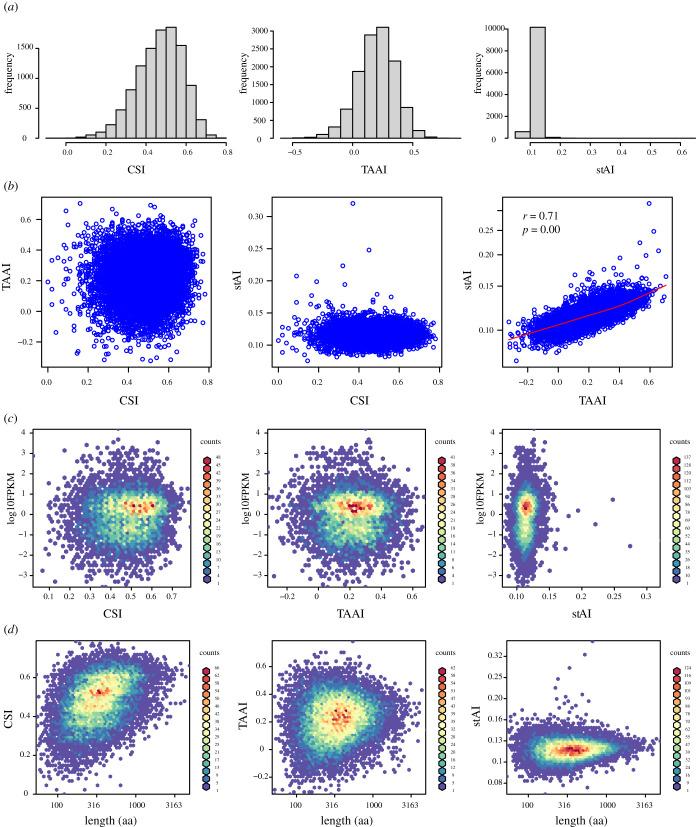
Co-adaption between tRNA gene frequency and codon usage at the synonymous codon level and the amino acid level of individual genes of *A. japonicus*. CSI: coefficient of synonymous codon usage to isoaccepting tRNA frequency; TAAI: tRNA gene copy number and amino acid usage accordance index [17]; stAI: tRNA adaptation index [18]. Data used in this figure can be found in electronic supplementary material, dataset S2. (*a*) Positive coefficients were observed for the majority of genes between codon usage and genomic tRNA content at the synonymous codon level (CSI), amino acid level (TAAI) and overall level (stAI). (*b*) The difference in adaptation in amino acid usage (TAAI) contributed the majority of the differences in overall adaptation of codon usage (stAI). (*c*) mRNA abundance was calculated as the average FPKM (fragments per kilobase per million mapped fragments) using transcriptome data from whole-body samples at 45 days, 75 days and nine months of age. mRNA abundance did not show a clear influence on codon usage adaptation. (*d*) Gene length has significant influences on codon usage adaptation.

To understand why codon usage adaption at the amino acid level was much lower than at the synonymous codon level, the correlation analysis was broken down to the individual gene level. For synonymous codon usage, the Pearson correlation coefficient of the synonymous codon frequency to the genomic isoaccepting tRNA frequency was calculated as in the last section (figure 2), but this time for individual genes instead of the whole transcriptome. The coefficient will hereafter be referred to as the CSI (coefficient of synonymous codon usage to isoaccepting tRNA frequency). For amino acid usage, the correlation was calculated as TAAI, namely the ‘tRNA gene copy number and amino acid usage accordance index’ [17]. As a result, positive correlations were observed for the majority of genes at both levels. Positive CSIs (median = 0.490) were found for 95.2% of *A. japonicus* genes while positive TAAIs (median = 0.215) were found for 90.8% of *A. japonicus* genes, indicating that most genes adapted to the genomic tRNA frequency in both their synonymous codon usage and amino acid usage (figure 5*a*).

To assess the contribution of synonymous codon usage and amino acid usage to the overall codon usage optimization, a third index was calculated to evaluate the overall level of codon usage adaptation. The species-specific tRNA adaptation index (stAI) was calculated for each gene, which considered codon usage at both the synonymous codon level and the amino acid level [19,20]. The coherence among the three indexes was evaluated (figure 5*b*). As a result, no correlation was observed between CSI and stAI, which indicated that usage of different synonymous codons for the same amino acid explained very little of the proportion of the differences in the overall level of codon adaptation. On the other hand, a significant positive correlation was found between TAAI and stAI (*R* = 0.71, *p* = 0), which indicated that the majority of the differences in codon usage optimization were derived from the usage of different amino acids.

To understand the variation of codon usage adaptation between individual genes, CSI, TAAI and stAI were compared with gene expression level and mRNA length. When all the genes were considered, no correlation was found between expression level with CSI, TAAI or stAI (figure 5*c*). Although the most abundantly expressed genes showed higher CSI than the genes with the lowest expression levels, the difference was small (electronic supplementary material, figure S2). A significant influence of mRNA length was observed for codon adaptation to tRNA content (figure 5*d*). As mRNA length increased, mRNAs with poor codon adaptation became scarce. At the same time, mRNAs with very high adaptation also became rare, especially for adaptation at the amino acid level (TAAI). The codon adaptation level seemed to converge to a constant as mRNA length increased.

**Figure 6.**
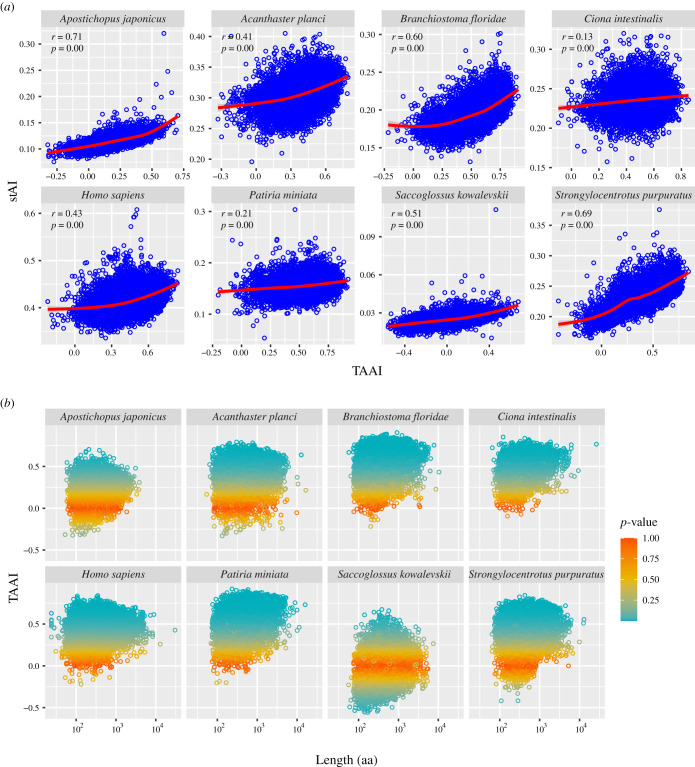
Correlation between TAAI, stAI and mRNA length in different deuterostomes. Data used in this figure can be found in electronic supplementary material, dataset S3. (*a*) A positive correlation was observed between TAAI (codon usage adaptation at the amino acid level) and stAI (overall codon usage adaptation level) for different deuterostomes. (*b*) The influence of gene length on codon usage adaptation is consistent among most deuterostomes.

To find out whether these observations were universal phenomena or specific to the sea cucumber, we then compared the distribution of TAAI and stAI among other echinoderms and deuterostomes. As a result, a positive correlation between TAAI and stAI was found for most of the species (figure 6). The influence of mRNA length on codon adaptation was also consistent among all the deuterostomes, as correlation between mRNA length and TAAI showed a triangle distribution in most cases.

### 2.4. Enzymes for catabolism, response to stimulus and saponin biosynthesis are adapted for efficient translation

Comparison between results in the last two sections exhibited that correlations between tRNA gene frequency and mRNA codon usage was much higher at the synonymous codon level (figure 2) than at the amino acid level (figure 4). However, it was the difference in amino acid usage that affected the overall level of codon usage adaptation (figure 6). A possible explanation was that optimization in synonymous codon usage had approached its maximum for most genes while amino acid usage had not. This is because using different synonymous codons for the same amino acid will not affect the function of the translated protein, but changes in amino acid usage of a protein will. Therefore, amino acid usage cannot be optimized in an unlimited way as for synonymous codons, but has to be a compromise between translation efficiency and protein functionality. Some genes may sacrifice their function for translation efficiency, while other proteins may do the opposite. To understand how the translation efficiency and functionality were balanced, we looked into amino acid–tRNA correlation for genes in different functional categories.

Enrichment analysis for Gene Ontology (GO) categories was carried out for genes with the highest (top 10%) and lowest (bottom 10%) TAAI (table 1). As a result, genes with the highest TAAI were enriched in ‘biological process’ categories including the catabolic process, signal transduction and response to stimulus. Genes enriched in the catabolic process include hydrolases of carbohydrate, lipid and peptide in the ‘molecular function’ category. The I-κB/NF-κB complex of the ‘cellular component’ category was enriched for high TAAI genes involved in signal transduction and response to stimulus. Many of the genes participating in signal transduction and response to stimulus were protein kinases that transfer phosphorus-containing groups. On the other hand, genes with the lowest TAAI were categorized as structural proteins of the ribosome, and transmembrane signalling receptors.

**Table 1.** GO categories enriched for genes with extreme TAAIs (GO level 3).

TAAI category	GO terms (level 3)	count	*p-*value	fold^a^	FDR^b^
top 10% (>0.40)	**biological process**				
	regulation of signal transduction	214	0.00	1.29	0.03
	regulation of cell communication	238	0.00	1.26	0.03
	organic substance catabolic process	200	0.00	1.27	0.05
	regulation of transcription factor activity^c^	39	0.00	1.79	0.07
	positive regulation of response to stimulus	141	0.00	1.29	0.08
	cell projection organization	149	0.00	1.28	0.08
	**cellular component**				
	extracellular space	96	0.00	1.46	0.04
	I-κB/NF-κB complex	5	0.00	7.85	0.17
	ciliary tip	10	0.00	3.27	0.19
	postsynaptic density	28	0.00	1.80	0.19
	**molecular function**				
	serine hydrolase activity	31	0.00	2.18	0.01
	cation binding	402	0.00	1.17	0.01
	hydrolase activity, acting on ester bonds	111	0.00	1.41	0.01
	protein–lipid complex binding	10	0.00	3.91	0.02
	peptidase activity	83	0.00	1.40	0.04
	apolipoprotein binding	8	0.00	4.17	0.04
	phosphorus-containing group transferase^d^	130	0.00	1.28	0.04
	hydrolase activity, acting on glycosyl bonds	26	0.00	1.92	0.04
bottom 10% (<0.037)	**biological process**				
	n.a.				
	**cellular component**				
	intrinsic component of membrane	474	0.00	1.28	0.00
	ribosomal subunit	38	0.00	2.46	0.00
	integral component of plasma membrane	137	0.00	1.49	0.00
	cell periphery	349	0.00	1.17	0.02
	receptor complex	35	0.01	1.60	0.20
	**molecular function**				
	transmembrane signalling receptor activity	152	0.00	2.50	0.00
	peptide receptor activity	35	0.00	3.85	0.00
	peptide hormone binding	13	0.00	3.30	0.01
	peptidase regulator activity	26	0.00	2.16	0.01
	sulfur-containing group transferase activity^e^	19	0.00	2.21	0.04
	photoreceptor activity	6	0.01	4.44	0.15

^a^Fold of enrichment.

^b^Benjamini–Hochberg false discovery rate.

^c^Short for ‘regulation of sequence-specific DNA binding transcription factor activity’.

^d^Short for ‘transferase activity, transferring phosphorus-containing groups'.

^e^Short for ‘transferase activity, transferring sulfur-containing groups’.

**Figure 7.**
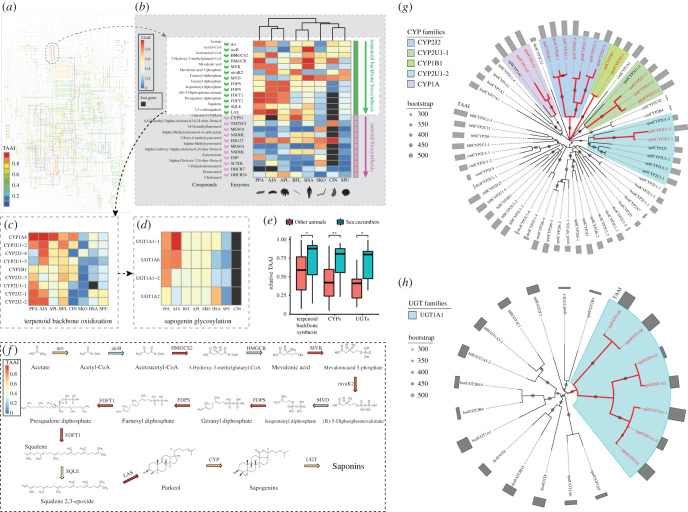
Enzymes in the saponin biosynthesis pathway of *A. japonicus* co-adapted with genomic tRNA content for efficient translation. Relative TAAI is calculated as the ranking of a gene's TAAI among all genes in the species. (*a*) Co-adaptation between amino acid usage in the metabolic pathway and genomic tRNA contents. The section for terpenoid backbone biosynthesis is encircled with a dashed line. A larger version of the figure can be found in electronic supplementary material, figure S3. (*b*) Translation adaptation in the pathway of terpenoid backbone biosynthesis and the downstream pathway of steroid biosynthesis. (AJA, *A. japonicus*; APL, *A. planci*; BFL, *Branchiostoma floridae*; CIN, *Ciona* *intestinalis*; HSA, *Homo* *sapiens*; PPA, *P. parvimensis*; SKO, *S. kowalevskii*; SPU, *S. purpuratus*). (*c*) Translation adaptation in sea cucumber-specific cytochrome P450 (CYP) gene families and their closest homologues. (*d*) Translation adaptation in sea cucumber-specific UDP-glycosyltransferase (UGT) genes and their closest homologues. Sequences of the CYPs and UGTs can be found in electronic supplementary material, dataset S4. (*e*) Comparison of translation adaptation in genes involved in saponin biosynthesis between sea cucumbers and other animal groups (two-tailed *t*-test for unequal variances). (*f*) Summary of the saponin biosynthesis pathway in *A. japonicus*. The enzyme catalysing each reaction is represented by an arrow dyed in the colour gradient according to its relative TAAI. (*g*) Phylogenetic analysis of sea cucumber-specific cytochrome P450 gene families and their closest homologues. Sea cucumber genes are shown by red lines and labels. Differences in branch lengths indicate that the sea cucumber genes evolved much faster than their homologues in other animals. (*h*) Phylogenetic analysis of sea cucumber-specific UGT gene families and their closest homologues. Sea cucumber genes are shown in red.

Enrichment analysis was also performed to map these genes into KEGG pathways (table 2). This revealed that high TAAI genes for ‘response to stimulus' in the GO category were mapped to KEGG pathways for response to virus or bacterial infection, including the cytosolic DNA-sensing pathway, Epstein–Barr virus infection, Toll-like receptor signalling pathway and NF-κB signalling pathway. To find hotspots optimized for translation efficiency in a large pathway such as the metabolic pathway, genes were visualized with a colour gradient according to their TAAI ranking. As a result, a section for the biosynthesis of secondary metabolites was highlighted with genes of high TAAI (electronic supplementary material, figure S3; figure 7*a*). A closer inspection revealed that this part of the metabolic pathway was known as the mevalonate (MVA) pathway (figure 7*b*), through which the terpenoid backbones of both sterols and triterpenes could be synthesized. For the sea cucumbers, the MVA pathway is essential for saponin synthesis [8,21]. TAAIs for enzymes in the MVA pathway were significantly higher than those of the rest of all genes of *A. japonicus* (*p*-value = 0.006, Wilcoxon rank-sum test). Terpenoid backbone biosynthesis is only the initial steps of saponin biosynthesis. In plants, the triterpenes are oxidized by specific cytochrome P450s (CYPs) to produce sapogenins, which undergo glycosylation catalysed by UDP-glycosyltransferases (UGTs) to produce saponins [22]. For sea cucumbers, the CYPs and UGTs for saponin synthesis are unknown. To single out candidate cytochrome CYPs for triterpene oxidation, we focused on CYPs identified as unique to sea cucumbers by our previous study [23]. Relative TAAIs of these CYPs in the two sea cucumber species is significantly higher than their homologous genes in other animal groups (figure 7*c*,*e*). Phylogenetic analysis confirmed that family CYP2J2 and CYP1B1 contains sea cucumber-only genes, while homologues of sea cucumber CYP2U1-1, CYP2U1-2 and CYP1A genes can still be recognized in the sea urchin or the starfish. Interestingly, in the latter case, the sea cucumber CYPs have been evolving much faster than their counterparts in the starfish or the sea urchin, as indicated by longer branch lengths (figure 7*g*). Thereby, we propose them as candidate genes for the oxidation of sea cucumber triterpenes. The same strategy was also used to identify UGTs that might be involved in the glycosylation of sea cucumber sapogenins. As a result, three of the four sea cucumber-specific UGT genes were highly optimized for translation (figure 7*d*,*e*), and the molecular distances to their homologues indicated accelerated evolution compared with the sea urchin or the starfish (figure 7*h*).

**Table 2.** KEGG pathways enriched for genes with extreme TAAIs.

TAAI category	KEGG class	KEGG pathway	genes	*p-*value	FDR
top 10%	infectious diseases	herpes simplex infection	22	0.00	0.02
	immune system	cytosolic DNA-sensing pathway	10	0.00	0.02
	immune system	toll-like receptor signalling pathway	10	0.00	0.07
	signal transduction	NF-κB signalling pathway	11	0.00	0.07
	immune system	IL-17 signalling pathway	10	0.00	0.07
	infectious diseases	measles	13	0.00	0.07
bottom 10%	translation	ribosome	51	0.00	0.00
	signalling molecules and interaction	neuroactive ligand–receptor interaction	20	0.00	0.00

### 2.5. tRNA gene frequency shows a correlation with particulate amino acids in coastal water

**Figure 8.**
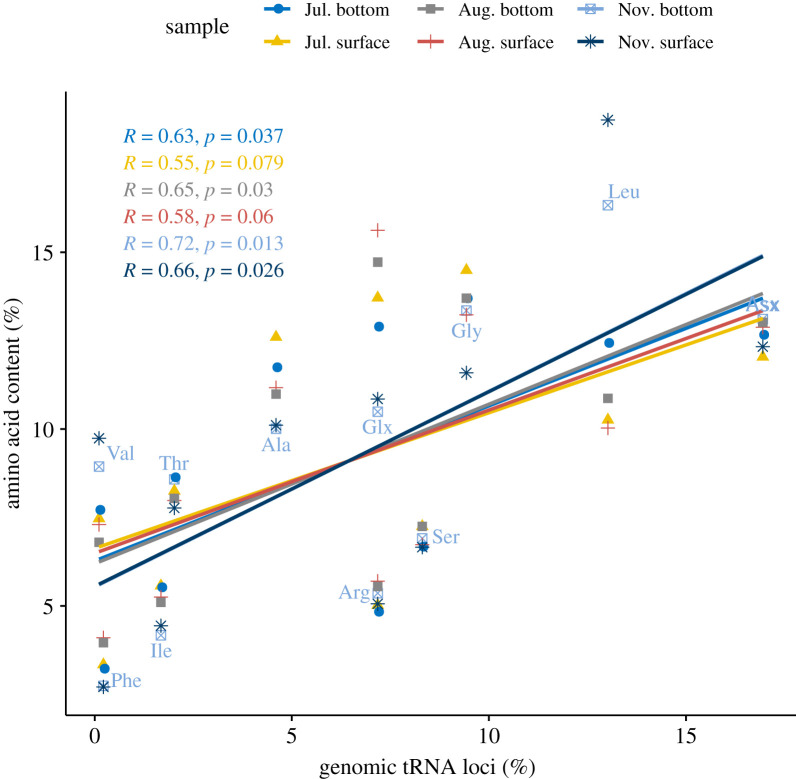
Correlation between tRNA gene numbers of *A. japonicus* and amino acid content of particulate organic matter in seawater on the east coast of China near the Yangtze Estuary.

Besides tRNA availability and mRNA codon usage, amino acid starvation may also influence the efficiency of mRNA translation [24,25]. Sea cucumbers feed by collecting marine debris deposited on the sea floor. The debris is poor in nutritional content, which may exert selective pressure on the organism's translation system. To investigate the relation between tRNA gene frequency and amino acid supply, we used data of the amino acid content in particulate organic matter in coastal waters, which were collected by Wu *et al.* [26] on the east coast of China near the Yangtze Estuary. A correlation analysis between amino acid content of the particles and the tRNA gene frequency of *A. japonicus* showed significant positive correlations (figure 8). Interestingly, the tRNA gene frequency showed a higher correlation with particles in the bottom seawater (which sea cucumbers inhabit) than those in the surface water, in each season (July: 0.63 versus 0.55; August: 0.65 versus 0.58; November: 0.72 versus 0.66). In addition, the tRNA gene frequency showed a higher correlation with amino acid content in particles available in the growing season (November) than in summer (July and August) when the sea cucumber goes into aestivation (0.66–0.72 versus 0.55–0.65).

## 3. Discussion

### 3.1. Why is tRNA expression correlated with genomic copy number

Unlike most other ncRNAs or coding genes, the cellular concentrations of tRNAs are highly correlated with their genomic copy numbers [4–6]. Our results here also support this theory, as high correlation was observed between codon usage and genomic tRNA content, which can only be explained by a relation between the tRNA gene number and the cellular tRNA abundance*.* Unlike other ncRNAs that reside within introns of protein-coding genes and form co-transcribed units with their host genes, tRNAs are devoid from intronic regions and tend to be transcribed independently (figure 1*b–d*). The reason for and significance of this characteristic have not been discussed before. We noted that the ncRNAs enriched in introns (i.e. miRNAs and snoRNAs) are those usually transcribed by RNA polymerase II (Pol II), which also transcribes the coding genes themselves [27]. On the other hand, the ncRNAs devoid from mRNA introns are those capable of being transcribed by Pol III (i.e. tRNAs and snRNAs) [27,28]. The intrinsic difference between the transcription mechanism of tRNAs and protein-coding genes may explain why tRNAs need to be transcribed independently. Pol III promoters differ significantly from Pol II promoters in that they are located downstream from the transcription start site and within the transcribed gene. Probably limited by the length and functionality of the gene, especially for a very short gene such as a tRNA, the intragenic promoters of Pol III are only regulated by the simple general transcription factors (GTFs), in particular TFIIIC2 and TFIIIB [29]. In fact, most tRNA promoters consist of an A box and a B box, which are highly conserved in tRNA genes from various organisms, probably in part because they encode the tRNA D- and T-loops, which are also required for tRNA function [29]. In other words, variation in the sequence of a tRNA promoter is restricted by the functionality of the tRNA itself. On the other hand, Pol II can interact with a bewildering array of extragenic transcription factors, which confers particular patterns of regulation [30,31]. Moreover, unlike coding genes, tRNA genes were not packaged into nucleosome particles that occlude them from interacting with transcription factors, suggesting a constitutive expression with little regulation at the chromatin level [32]. In brief, the limited capacity of short tRNA genes to contain complicated intragenic regulatory sequences, together with the absence of regulation at the chromatin level, may explain why cellular tRNA pools are simply determined by tRNA gene copy numbers. The same reasons may also explain why an organism needs so many tRNA gene copies that make tRNAs the most numerous ncRNAs in a genome. In fact, if the genome of an organism is too small to accommodate a reasonable number of tRNAs, selection on codon usage becomes impossible [20].

### 3.2. Efficient translation is crucial for transiently expressed genes

**Figure 9.**
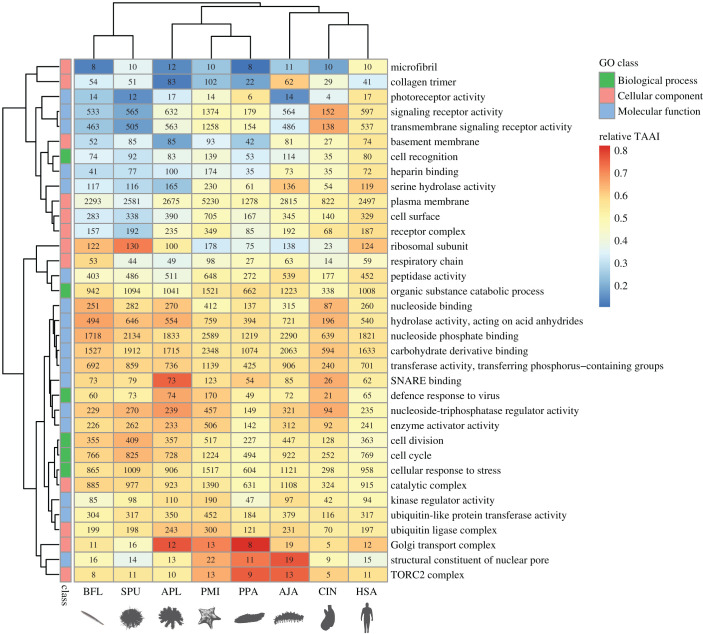
Comparison of co-adaptation between amino acid usage and genomic tRNA contents among gene categories across different deuterostomes. Relative TAAI (ranking of a gene's TAAI among all genes in the species) was averaged among genes in a GO category. Numbers in the heat map represent the number of gene families belonging to each GO category. (AJA, *A. japonicus*; APL, *A. planci*; BFL, *B. floridae*; CIN, *C.* *intestinalis*; HSA, *H.* *sapiens*; PMI, *P. miniata*; PPA, *P. parvimensis*; SPU, *S. purpuratus*).

Recently, there has been increasing evidence that the degree of codon optimization is different among cell types and species [1]. Previous studies have suggested that proteins connected to acute responses have higher co-adaption intensity [17,33]. In this study, we also found that genes regulating ‘signal transduction’ during ‘response to stimulus' in the ‘immune system’ were intensely optimized for translation efficiency (table 2). Considering that the translation and degradation of these proteins controls the persistence of the immune response, they need to be produced promptly in response to pathogen infection, and then be degraded when the stress is over, otherwise they will be harmful to the host itself. The scenario is similar for enzymes such as peptase and hydrolase, which participate in the ‘organic substance catabolic process' (table 1). They have to be secreted quickly when food becomes available and then will be expended when they are mixed with food and go through the intestine. To assess whether this pattern is specific to the sea cucumber or universal, we extended TAAI analysis to other echinoderms and deuterostomes. GO categories were compared among species according to the average TAAI of their genes. As a result, the pattern is consistent among most animals (figure 9). Genes that are the most optimized for translation efficiency include those in the stress-sensing TORC2 complex and those involved in exocytosis and secretion (SNARE binding, Golgi transport complex). On the other hand, structural proteins in the microfibril, collagen trimer, basement membrane and signalling receptors are least optimized in codon usage. A seeming exception is the ‘structural constituent of nuclear pore’, which shows high translation efficiency. This may be explained by the fact that nuclear pore complexes (NPCs) are dynamic structures, whose number increases dramatically in preparation for cell division and in response to hormone stimulation [34,35]. In general, we speculate that proteins with high turnover rates need to be translated more quickly, thus are more optimized for codon usage. We searched for existing data to support this hypothesis, and a study on protein half-lives provided positive evidence [36], as the protein groups found to have high turnover rates matched the high TAAI groups found in the present study (electronic supplementary material, table S2). Another work on translation elongation speed also found that genes with low translation speed encoded more stable proteins [37].

Our study also showed that mRNA length significantly affected codon usage adaptation. As mRNA length increased, mRNAs with very poor adaptation or very high adaptation both became scarce, and the codon adaptation level seemed to converge to a constant (figures 5*d* and 6*b*). This phenomenon seems contradictory, but both sides of the story may have an explanation. The absence of very long genes with a very high adaptation level is consistent with a previous observation that shorter genes have a higher level of codon usage adaptation than longer genes [38]. This observation fits in the context of our discussion above that genes with a very high codon adaptation level are those that need to be translated quickly, so a very long mRNA is disqualified from participating in such an urgent response because it cannot be translated quickly enough. On the other hand, genes with a very poor codon adaptation level tend to be very short. This is probably because very short genes lack codon redundancy, which makes codon usage adaptation to tRNA gene frequency difficult. In this case, codon usage is constrained by a lack of codon redundancy and the functionality of the protein. However, functional amino acid sequences of some of the short genes ‘unfortunately’ contradict the genomic tRNA gene proportion, and they end up with a low codon adaptation level. Figure 6*b* shows that, in most of the species, low TAAIs of the short genes have high *p-*values, which means that the low TAAIs (coefficients) are not statistically significant. This observation supports the above hypothesis that most of the low codon usage adaptations of short genes are random events.

### 3.3. Optimized translation in saponin biosynthesis

Sea cucumbers are special in the animal kingdom in that they produce triterpenoid glycosides (i.e. saponins), which are commonly synthesized by plants but are only found in a few animal lineages. Saponins can interact with sterols in the membrane, which changes membrane stability, microviscosity and permeability. These membranolytic interactions are especially poisonous to fish, so saponins provide sea cucumbers with a defence mechanism against predators [39]. The way in which sea cucumbers obtain a plant-like ability to synthesize saponins is only partially understood. In plants, the triterpene skeletons of saponins are synthesized via the mevalonate (MVA) pathway. The triterpenes are then oxidized into sapogenins, which undergo glycosylation to produce saponins [22]. Although it is agreed that sea cucumbers can produce sapogenins from 2,3-oxidosqualene, whether the latter is produced by de novo synthesis through the MVA pathway or obtained from dietary sources is still under debate [39]. Our results here reveal that codon usage of key enzymes in the MVA pathway is highly optimized for efficient translation, suggesting that the sea cucumber can produce 2,3-oxidosqualene by itself. In plants, oxidization of triterpenes is catalysed by specific cytochrome P450s (CYPs) [22]. It was unknown which of the numerous CYPs performed the same role in sea cucumbers. To single out candidate CYPs for saponin biosynthesis, we focused on CYPs that are unique to sea cucumbers because sea cucumber saponins are intrinsically different from those of plants or starfish [39], so CYPs for their synthesis might also be unique. We found that sea cucumber-specific CYPs underwent intense selection for translation efficiency and evolved faster than their counterparts in other echinoderms. We propose these CYPs as candidate genes for the oxidation of sea cucumber triterpenes. We also identified three sea cucumber-specific UGTs as candidates for glycosylation of sapogenins. In brief, we suggest that sea cucumber genes in the entire pathway of saponin biosynthesis have been under strong selection for efficient translation, which may increase saponin production. This finding is consistent with a previous discovery that a sea cucumber gene (LAS) for saponin synthesis shows high evolutionary rates compared with other diverse animal groups, and contains many plant-like motifs that are not present in sea urchin and starfish [21]. Nevertheless, the functions of these sea cucumber CYPs and UGTs in saponin biosynthesis need to be experimentally verified in future studies.

### 3.4. Adaptation to available amino acid resource

Besides tRNA availability and mRNA codon usage, the availability of amino acids may also affect the efficiency of translation. Conditions in which the amino acid supply is limiting to growth were found to result in changes in tRNA charging levels [24,25], causing some normally suboptimal codons to become translationally optimal. For sea cucumbers, which feed on marine sediment that is very poor in nutritional content, this may provide selective pressure on the organism and affect its translation system. We found that the tRNA content of *A. japonicus* was positively correlated with amino acid content in the food particles (figure 8). Interestingly, the tRNA gene frequency showed a higher correlation with particles in the bottom seawater (which sea cucumbers inhabit) than with those in the surface water, and the correlation was higher for food resources available in the growing season than in the aestivation season. Whether the genomic tRNA content of the sea cucumber has evolved to meet the available food resource demands further investigation. However, the consistency of amino acid intake and the cellular tRNA repertoire should help the sea cucumber synthesize its own proteins more efficiently and minimize resource/energy costs. For animals living on a food resource with very low nutritional content, this may be critical for their survival.

## 4. Conclusion

Using the sea cucumber as a model, we combined genomic sequences, transcriptome expression and ecological food resource data to study its codon usage adaptation. Our analyses revealed that codon usage of enzymes catalysing saponin biosynthesis and responses to stimuli were highly optimized for efficient translation, which may facilitate the synthesis of saponins as defensive chemicals and promote survival under external stresses. Moreover, enzymes for digestion were also optimized for efficient translation, and the tRNA repertoire matched the amino acid content in the natural food particles of *A. japonicus*, which should help it to survive on marine debris with very low nutritional content. The finding from bioinformatics analysis in this work should serve as a launchpad for further experimental research.

## 5. Material and methods

### 5.1. tRNA gene annotation

For de novo annotation of tRNA genes, genome assemblies of the following species were retrieved from the NCBI genome database: *A. japonicus*, *Acanthaster* *planci*, *D. rerio* and *S. kowalevski*. Genome assemblies of several echinoderms were retrieved for tRNA annotation from EchinoBase (echinobase.org), including *Lytechinus variegatus*, *Patiria miniata* and *Parastichopus parvimensis*.

Identification of genomic tRNA is usually hampered by repetitive elements, because short interspersed nucleotide repetitive elements (SINEs) are difficult to distinguish from tRNAs. Two strategies were tried to solve this problem for prediction of *A. japonicus* tRNA genes. A post-filtering strategy suggested by GtRNAdb was carried out, using tRNAscan-SE 2.0 (lowelab.ucsc.edu/tRNAscan-SE) [40], with the legacy mode and requiring identification with both tRNAscan and EufindtRNA. Then a pre-filtering with RepeatModeler and RepeatMasker (-norna) was also tested, followed by tRNAscan-SE. Comparison between the results showed consistent results for most of the tRNAs. However, the post-filtering strategy produced exceptionally high copies of Cys and Tyr for *A. japonicus* (electronic supplementary material, figure S1). Further investigation of transcriptome codon usage indicated that the exceptionally high copies of Cys and Tyr are unlikely to be real tRNA genes but repetitive elements, so the pre-filtering strategy was adopted as the chosen method. The strategy was then used for de novo identification of tRNAs for other species.

tRNA annotations of the following species were downloaded from the Genomic tRNA Database (GtRNAdb) [2], which also used tRNAscan-SE: *Branchiostoma floridae*, *Ciona* *intestinalis*, *Homo sapiens* and *Strongylocentrotus purpuratus*.

### 5.2. Coding sequences

mRNA and protein sequences of *A. japonicus* were obtained in our previous work [23]; these are available from the sea cucumber genome database (genedatabase.cn/aja_genome_20161129.html). Protein and CDS sequences of the following species were retrieved from the NCBI genome database: *A. planci*, *B. floridae*, *C. intestinalis*, *H. sapiens*, *S. kowalevskii* and *S. purpuratus*. Protein-coding sequences of several echinoderms were retrieved from EchinoBase (echinobase.org), including *L. variegatus*, *P. miniata* and *P. parvimensis*. Specifically, for species with any gene missing in the mevalonate (MVA) and steroid biosynthesis pathways, the original gene annotations were verified by BLAST search against the genome, followed by Genscan prediction of gene structure.

### 5.3. Expression data

To obtain expression information for CDS, transcriptome sequencing reads of *A. japonicus* were retrieved from the NCBI SRA database, including samples of whole body at 45 days, 75 days and nine months of age (SRA entry SRX3196455, SRX3196424 and SRX3763489). The reads were aligned to the genome and transcripts were assembled using HISAT2 and StringTie. All the previously annotated transcripts were recovered. The expressions of transcripts were calculated as fragments of transcript per million mapped reads (FPM) using Ballgown [41].

### 5.4. Amino acid content in particulate organic matter of seawater

To investigate the relation between tRNA gene frequency and amino acid supply, data for amino acid content in particulate organic matter of coastal seawater were obtained from the work of Wu *et al*. [26], who collected samples from the east coast of China near the Yangtze Estuary [26]. For the determination of particulate amino acids (PAAs), the original work used HCl to hydrolyse the particles and a high-performance liquid chromatography system to fluorometrically determine the individual amino acids in the hydrolysates.

### 5.5. Correlation between isoacceptor tRNA gene content and synonymous codon usage

For correlation analysis between isoacceptor tRNA copy number and synonymous codon usage, the genomic frequency of the *j*th tRNA among the *n_i_* isoacceptor tRNAs that translate amino acid *i* was calculated as

5.1 $${\mathrm{TF}}_{ij}=\frac{t_{ij}}{\sum_{\;j=1}^{n_{i}}t_{ij}},$$

where *t_ij_* is the genomic copy number of tRNA *ij*.

For an individual gene, the frequency of the *j*th codon among the *n_i_* synonymous codons that encode amino acid *i* of an mRNA was calculated as

5.2 $${\mathrm{CF}}_{ij}=\frac{c_{ij}}{\sum_{\;j=1}^{n_{i}}c_{ij}},$$

where *c_ij_* is the number of codon *ij* in a mRNA.

For all the *m* genes of *A. japonicus*, the frequency of the *j*th codon among the *n_i_* synonymous codons that encode amino acid *i* was calculated as

5.3 $${\mathrm{TCF}}_{ij}=\frac{\sum_{k=1}^{m}e_{k}c_{ijk}}{\sum_{\;j=1}^{n_{i}}\sum_{k=1}^{m}e_{k}c_{ijk}},$$

where *c_ijk_* is the number of codon *ij* in the *k*th gene. *e_k_* is the expression level (FPM or fragments per million reads) of the *k*th gene, averaged among whole body transcriptomes at 45 days, 75 days and nine months of age. The reason for using FPM instead of FPKM (fragments per kilobase per million mapped fragments) for gene expression is that FPM contains the length information of a transcript. Because a long transcript can be translated at multiple positions along its length at the same time, it can interact with multiple tRNAs simultaneously [42]. As we will discuss correlation between mRNA codon usage and tRNA availability, the length information should be kept in the calculation. For multiple transcripts encoded by the same gene via alternative splicing, sequencing reads were equally allocated among the transcripts during expression calculation.

Correlation between isoacceptor tRNA copy number and synonymous codon usage was then calculated as the Pearson coefficient of TF*_ij_* versus CF*_ij_* or TF*_ij_* versus TCF*_ij_*. For convenience, the coefficient for individual genes (TF*_ij_* versus CF*_ij_*) was referred to as the CSI (coefficient of synonymous codon usage to isoaccepting tRNA frequency).

Comparisons between isoacceptor tRNA gene content and synonymous codon usage were calculated using two different rules. Basic correlations were calculated by using the classical Watson–Crick base pairing rules (U : A; A : U; C : G; G : C), while extended correlations were calculated allowing wobble base pairing at position 34 of tRNA, including G : U wobble and results from the activities of ADATs (I : A; I : C; I : U). For extended correlation calculations, codons were equally allocated among corresponding alternative anticodons.

### 5.6. Correlation between tRNA gene content and amino acid usage

To assess the co-adaption between tRNA gene copy number and amino acid usage of a coding sequence, an index was calculated as the Pearson correlation coefficient between amino acid usage of an individual gene and tRNA gene copy number of the genome. The measure was proposed by Du *et al*. [17] as the ‘tRNA gene copy number and amino acid usage accordance index (TAAI)’ [17].

For transcriptome-level analysis, the Pearson coefficient was calculated between tRNA gene copy number and accumulated amino acid count of all the genes of *A. japonicus.* During accumulation of the amino acid count, each amino acid in each gene was weighted with the gene's expression level (FPM) averaged among whole body transcriptomes at 45 days, 75 days and nine months of age.

### 5.7. tRNA adaptation index

The tRNA adaptation index (tAI) is also a measure of the efficiency by which a coding sequence is recognized by the intracellular tRNA pool [20]. Unlike CSI, which considers only synonymous codon usage, or TAAI, which considers only amino acid composition, tAI contains codon usage information at both levels. In this study, we adopted an upgraded version of tAI called species-specific tAI (stAI), which was calculated using stAIcalc [18,19].

### 5.8. Enrichment analysis for gene ontology and KEGG pathways

Genes with the highest (top 10%) or lowest (bottom 10%) TAAI were categorized using GO and KEGG pathway enrichment analysis (*p*-value ≤ 0.01), carried out using DAVID (david.ncifcrf.gov) and omicshare (omicshare.com/tools), respectively.

## Data accessibility

The datasets used and/or analysed during the current study are available as online supplementary data and/or from the corresponding author on reasonable request. The data are provided in the electronic supplementary material [43].
